# Notes on Medicine for Medical and Dental Students

**Published:** 1902-11-22

**Authors:** 


### Editor's Letter-Box.

[Our Correspondents are reminded that prolixity is a great bar to publication, and that brevity of style and conciseness of statement greatly facilitate early insertion.]

### "NOTES ON MEDICINE FOR MEDICAL AND DENTAL STUDENTS."

Mr. W. D. Woodburn, of Greenock, writes:—Acting under advice, I beg to draw your attention to the review of a small book I published entitled "Notes on Medicine for Medical and Dental Students."

I may frankly say that your reviewer has a perfect right to say what he pleases provided he does so on fair grounds; but in this case he has gone a little way beyond his bounds, and trespassed in such a manner as to be an injustice.

I admit many serious errors which were not explained in preface, for the reason they could not be, as I never had the opportunity of correcting them myself, being unwell and confined to bed after the proofs had been started in type and I could not withdraw the issue. Probably this may be no excuse for the many errors; nevertheless I give it.

But the point to which I feel an injustice has been done is evidently on the ground of a misunderstanding on his part, and it reads very like as if the action was intended to injure permanently a small attempt on my part to contribute to the help of others. It is unfair to have said that I presumed to act the part of "teacher" on a subject that I would not for an instant dare to talk upon myself, and the last few sentences try to make out that I am the "authority," which is not the case, but the lecturer from whom the notes were taken. Personally I know not whether one thing is better than another for this trouble, not having treated such a case, but still the note is from a teacher on the subject, *not myself.* I had no desire, when I published this little volume, to gain anything by it, either from a pecuniary point or self-glorification, but merely, as I say in preface, to give to my fellow students, in book form, notes I had collected, hoping it might be of use to them, and instead of getting a little encouragement from your reviewer, who at least might have had some slight consideration, he gives it such a hammering as to make it impossible to live. Your mandate has gone forth, so I need not attempt to expect a reprieve; but I did not anticipate such treatment at the hands of The Hospital.

[In regard to the lapses which are admitted, it is unnecessary to say anything. Mr. Woodburn's chief complaint appears to hinge upon the point that no mention is made of the fact that the "Notes" represent the views of the teacher rather than of the note-taker by whom they are published, and there might be something in this contention were there anything in the title, in the preface, or in the contents of the book to suggest that it consisted of notes of any particular course of lectures by any individual lecturer. This, however, is not the case. The preface says, "I claim no originality for these notes, which were collected and arranged from all quarters, viz., class notes, from lectures delivered by Sir William Gardiner (*sic*), Professor Samson Gemmell, and from the text-books of Fagge, Payne, Powell, Mackenzie, Crocker, Morris, Taylor, etc." Now, when a writer dips into the thousands of pages represented by the above works—practically into the general well of human knowledge—and produces 76 small pages of notes it is surely not too much to say that that writer must be held responsible for the opinions set forth, unless in each case he gives his authority. It is idle to say, "The note is from a teacher on the subject, not myself." What teacher? As a matter of fact, in the particular note referred to, the names of three teachers are given in regard to special modes of treating aneurysm, but the summing up, the judgment, the statement which treatment "is best" is made anonymously. For such a statement the author cannot throw over his responsibility.—The Writer of the Review.]

